# Multidimensional mechanisms and therapies underlying gastroesophageal reflux disease: focus on immunity, signaling pathways, and the microbiota-gut-brain axis

**DOI:** 10.3389/fimmu.2025.1629944

**Published:** 2025-07-18

**Authors:** Jiajing Zheng, Lin Tao

**Affiliations:** Department of Digestive, Beijing Hospital of Traditional Chinese Medicine Affiliated to Capital Medical University, Beijing, China

**Keywords:** gastroesophageal reflux disease, lower esophageal sphincter, immune cells, cytokines, NF-κB, MAPK, PI3K-Akt, microbiota-gut-brain axis

## Abstract

Gastroesophageal reflux disease (GERD) has a high incidence rate and a complex pathogenesis that is not yet fully understood. This review aims to provide a comprehensive exploration of the mechanisms underlying GERD, emphasizing the interplay between immune responses, signaling pathways, and the microbiota-gut-brain axis. Specifically, it highlights the contributions of immune cells (e.g., T-lymphocytes, dendritic cells, mast cells), pro-inflammatory cytokines, and key signaling pathways, including nuclear factor (NF)-κB, mitogen-activated protein kinase (MAPK), and phosphoinositide 3-kinase (PI3K)/protein kinase B (Akt), in driving esophageal inflammation and barrier dysfunction. Furthermore, the review examines the bidirectional interactions between psychological stress, gut microbiota dysbiosis, and GERD pathophysiology via the gut-brain axis. In bridging these mechanisms to potential therapeutic strategies, this review evaluates both established pharmacological treatments, such as proton pump inhibitors (PPIs) and immunotherapy, and emerging approaches, including herbal formulations and neuromodulation techniques. By synthesizing current evidence, the review identifies critical knowledge gaps, particularly in understanding the cross-talk between immune pathways and therapeutic targets. These findings underscore the need for mechanism-driven research to facilitate the development of personalized treatment strategies and address unresolved challenges in GERD management.

## 1 Introduction

Gastroesophageal reflux disease (GERD) is a chronic and multifactorial disorder characterized by the reflux of gastric contents into the esophagus, leading to mucosal damage and a range of clinical symptoms (1). Based on endoscopic findings, GERD can be categorized into Barrett’s esophagus (BE), reflux esophagitis (RE), and non-erosive reflux disease (NERD). The pathogenesis of GERD is primarily related to reduced pressure of the lower esophageal sphincter (LES), which leads to reflux of gastric contents into the esophagus, causing mucosal damage and various clinical symptoms. A burning sensation behind the sternum that radiates to the neck, throat, and occasionally the back is a typical signs of GERD. Some patients may also experience dysphagia, chest discomfort, and a persistent cough (2). If left uncontrolled for an extended period, GERD may lead to esophagitis and esophageal ulcers and may even cause cellular changes that increase the risk of BE, considerably raising the risk of esophageal adenocarcinoma (3). However, GERD is increasingly recognized as a multifactorial disorder, extending beyond acid-related mechanisms. In addition to LES dysfunction, emerging evidence implicates immunological dysregulation, visceral hypersensitivity, neuromotor dysfunctions, and MGBA interactions in disease development. Currently, proton pump inhibitors (PPIs) are considered the first-line treatment for GERD, as they have been shown to relieve symptoms and promote mucosal healing by inhibiting gastric acid secretion. However, studies have shown that up to 40% of patients with suspected GERD do not experience adequate symptomatic relief after taking PPIs, a phenomenon known as “refractory GERD” (4, 5). While PPIs remain a cornerstone of GERD treatment due to their acid-suppressive and anti-inflammatory effects, their limitations highlight the need to address non-acid-related contributors. From an epidemiologic point of view, the prevalence of GERD is significantly increasing worldwide. From 1990 to 2019, the overall number of people with common GERD rose by 77.53% (6). However, the incidence of GERD varies significantly by geography. Approximately 10–20% more people in Western nations have GERD than in other countries. In contrast, the prevalence in Asian countries is relatively low, usually less than 10% (7). The underlying pathogenesis of GERD involves LES dysfunction, immune cell-mediated inflammation, abnormal activation of signaling pathways and microbiota-gut-brain axis (MGBA) dysregulation. By integrating advances in immunology, molecular biology, and neurogastroenterology, this review aims to provide an updated and comprehensive overview of GERD mechanisms and identify novel therapeutic targets.

## 2 Methodology

This review was conducted through a systematic search of articles published between 2014 and 2024 in PubMed, Google Scholar, and Web of Science databases. To ensure the inclusion of foundational and influential studies, a limited number of articles published between 2000 and 2013 were also incorporated. The selection process emphasized relevance to the topic, with a focus on studies that provided experimental validation, clinical relevance, or mechanistic insights. The search strategy utilized keywords such as “GERD pathogenesis,” “GERD and lower esophageal sphincter,” “GERD and immune cells,” “GERD and T-lymphocytes,” “GERD and mast cells,” “GERD and dendritic cells,” “GERD and cytokines,” “GERD and signaling pathways,” “GERD and NF-κB signaling pathway,” “GERD and MAPK signaling pathway,” “GERD and PI3K-Akt signaling pathway,” “GERD and microbiota-gut-brain axis,” and “GERD and psychiatric disorders.” Articles were selected based on their relevance, originality, and timeliness. The inclusion criteria focused on studies investigating the roles of immunity, signaling pathways, and the gut-brain axis in the pathogenesis of GERD, as well as research exploring novel therapeutic approaches. Priority was given to studies involving animal models, clinical trials, and molecular mechanisms. Articles that lacked experimental validation or clinical relevance were excluded. A total of 109 articles were referenced in this review. **Table 1** presents the study designs, which exclusively include research with clearly defined methodologies. These studies incorporate both human participants and rat animal models. The included research investigates various therapeutic approaches, including Non-Drug Therapy, Drug Therapy, Biological Therapy, Traditional Chinese Medicine Therapy, Natural Medicine Therapy, Neurostimulation Therapy, and Acupuncture Therapy. Key aspects evaluated in these studies encompass Efficacy, Dosage and Duration, Selection Criteria, Regions of Action, Pathways of Action, and Targets of Action. This comprehensive approach ensures a systematic analysis of the methodologies and outcomes across diverse therapeutic interventions.

**Table 1:** Efficacy and mechanisms of therapeutic modalities for GERD.

Treatment type	Treatment name	Research model	Dosage and duration	Selection criteria	Efficacy	Regions of action	Pathways of action	Targets of action	Reference
Non-Drug Therapy	Anti-reflux mucosal resection	Clinical (Human)	Resection of EGJ circumference by 50%, extending 2 cm into the cardia. Single intervention with post-procedure follow-up over a period	Patients diagnosed with GERD. Persistent symptoms despite twice-daily use of PPI.	Reduced the number of reflux episodes, shortened the duration of acid reflux into the esophagus, and inhibited TLESR.	Esophago-gastric junction, esophageal mucosa.	Inhibition of TLESR by mucosal resection reduces the sensitivity of gastric tract receptors.	LES, TLESR, gastric stretch receptor, esophageal mucosal barrier.	(8)
Non-Drug Therapy	Lower esophageal sphincter electrical stimulation	Clinical (Human)	Electrical stimulation parameters set at 5 mA, 20 Hz, and 220 μs pulse width. 1-month follow-up.	PPI-refractory GERD patients with IEM	Demonstrated safety and efficacy in patients with GERD combined with IEM, with no effect on swallowing function at 1 month postoperatively.	LES	Enhancement of the contractile function of the LES by electrical stimulation increases the pressure of the anti-reflux barrier and reduces acid reflux.	LES, esophageal motor function.	(9)
Drug Therapy	PPI	Clinical (Human)	40 mg/d, lasted 1 month	Patients with upper gastrointestinal tract symptoms and GERD diagnosed through endoscopy and histopathology	Inhibited gastric acid secretion, significantly reduced T-cell counts, and alleviated the inflammatory response in 66% of patients, but still resulted in inflammatory resistance due to persistent T-cell activation in 33% of patients.	Immune system, esophageal mucosa	Inhibition of gastric acid secretion indirectly reduces the esophageal mucosa’s inflammatory response. Immune suppression reduces the number of T cells in the peripheral blood. Reduces IL-8 and IL-1β levels.	CD3^+^, CD4^+^, CD8^+^, IL-8, IL-1β	(10)
Biological Therapy	Immunotherapy with DCs	Clinical (Human)	Once every 3 weeks, a total of 4 times	Late stage EAC patients	In combination with chemotherapy, radiotherapy, or targeted therapy, it can synergistically enhance the anti-tumor immune response and improve the clearance of tumor cells.	Immune system and immune cells in the local tumor microenvironment	Enhances anti-tumor immune response by DCs loaded with TAAs, activating CTLs. Combined with chemotherapy, radiotherapy, or targeted therapy, it synergistically enhances immune response.	DCs, TAAs, CTLs, anti-VEGF drugs, anti-HER-2/neu drugs	(11)
Traditional Chinese Medicine Therapy	Hewei Jiangni granules	Rat model	High dose: 4.3 g/kg/d. Medium dose: 2.2 g/kg/d. Low dose: 1.4 g/kg/d. Comparison group: Omeprazole 8.4 mg/kg/d. Daily intragastric administration for 14 days	NERD rat models established through basal sensitization and acid infusion.	Reduced the esophageal mucosal cell gap, attenuated esophageal mucosal injury, and relieved NERD symptoms.	Esophageal mucosa, nervous system	Regulates TRPV1 and TRPM8 channels, inhibits degranulation of MCs, and reduces the release of neurochemical substances (SP, CGRP, PAR2).	MCs, TRPV1, TRPM8, SP, CGRP, PAR2	(12)
Traditional Chinese Medicine Therapy	Jianpi Qinghua granules	Clinical (Human)	Take one packet of medicine three times a day for a total of 4 weeks	Diagnosed with NERD characterized by spleen deficiency and damp-heat syndrome.	Improved reflux and heartburn symptoms with an improvement rate of 79.49%. Improved spleen deficiency and damp-heat syndrome. Improved GERD-HRQL. Decreased self-assessed depression and anxiety scores.	Gastrointestinal, nervous system	Promotes proliferation and activation of MCs and reduces sensitization of nerve endings.	MCs, nerve endings	(13)
Natural Medicine Therapy	Berberine	Rat model	20, 40, and 60 mg/kg. Rats were treated with BB 1 hour before surgery and sacrificed 6 hours after surgery for evaluation.	A rat model of GERD induced by pylorus and forestomach ligation.	Reduced inflammatory response and exhibited low cytotoxicity.	Immune system, esophageal mucosa	Activates the AMPK pathway. Inhibits the release of inflammatory cytokines.	AMPK, TNF-α, IL-1β, IL-6, MCP-1, PAI-1	(14)
Natural Medicine Therapy	Fermented soybean	Clinical (Human)	1g/d, lasted 12 weeks	Adults diagnosed with GERD.	Significantly improved heartburn and reduced reflux symptoms.	Immune system, gastrointestinal system	Modulates the inflammatory response through anti-inflammatory effects	IL-8, IL-6, IL-4	(15)
Traditional Chinese Medicine Therapy	Rhei Rhizoma	Rat model	125 or 250 mg/kg/day for 7 days	Healthy rats suitable for surgical induction of reflux esophagitis.	Eliminated heat, purged fire, activated blood circulation, dispersed blood stasis	Immune system, esophageal mucosa	Activates the Nrf2/HO-1 pathway, inhibits the MAPK signaling pathway, and blocks NF-κB nuclear translocation.	Nrf2/HO-1, MAPK, NF-κB	(16)
Natural Medicine Therapy	Geraniin	Rat model	15 and 30 mg/kg/day. Single-dose administration 90 minutes before surgery	Healthy rats suitable for reflux esophagitis induction	Showed antioxidant and anti-inflammatory effects.	Immune system, esophageal mucosa.	Inhibits the NF-κB and Nrf2 signaling pathways and reduces macrophage inflammatory responses.	NF-κB, Nrf2, macrophage.	(17, 18)
Natural Medicine Therapy	Quercetin	Rat model	100 and 200 mg/kg body weight. Lasted for 6 weeks	Rats suitable for surgical induction of chronic mixed RE.	Showed antioxidant, anti-inflammatory, antiviral, and immunomodulatory effects. Prevented esophageal mucosal injury in RE rats.	Immune system, esophageal mucosa	Inhibits the NF-κB p65 and IL-8 signaling pathways and reduces inflammatory responses.	NF-κB p65, IL-8	(19, 20)
Traditional Chinese Medicine Therapy	Zuojin Pill	Rat model	Low-dose: 0.63 g/kg/day. Medium-dose: 1.26 g/kg/day. High-dose: 2.52 g/kg/day. Lasted for 4 weeks.	Rats capable of undergoing esophagogastric anastomosis and suitable for therapeutic interventions.	Reduced pro-inflammatory cytokine levels and enhanced esophageal mucosal barrier integrity.	Immune system, esophageal mucosa	Inhibits the MAPK/NF-κB signaling pathway and reduces the production of TNF-α, IL-6, and IL-1β.	MAPK, NF-κB, TNF-α, IL-6, IL-1β	(21)
Natural Medicine Therapy	Atractylenolide III	Rat model	Low-dose: 0.6 mg/kg/day.;High-dose: 2.4 mg/kg/day. Lasted for 28 days.	Rats capable of undergoing the sequential surgical procedures to induce RE.	Reduced oxidative stress and inflammatory responses.	Immune system, esophageal mucosa	Inhibits the PI3K/AKT/NF-κB/iNOS signaling pathway.	PI3K/AKT, NF-κB, iNOS	(22)
Traditional Chinese Medicine Therapy	Zhizhu Pill	In silico study (using network pharmacology and molecular docking)	Not applicable	Active compounds of ZZP were identified based on available data in public compound databases. GERD-related target genes were selected from public gene databases.	Reduced oxidative stress and inflammatory responses.	Immune system, esophageal mucosa	Inhibits the PI3K/AKT signaling pathway.	PI3K/AKT	(23)
Traditional Chinese Medicine Therapy	Shugan Jiangni Hewei granules	Rat model	4 g/kg/day. Lasted for 14 days.	Rats capable of undergoing induction of visceral hypersensitivity to mimic NERD.	Showed neuroprotective effects and improved visceral hypersensitivity.	Immune system, esophageal mucosa.	Regulates the PI3K/Akt signaling pathway and reduces Pik3r2 expression.	PI3K/Akt, Pik3r2.	(24)
Neurostimulation Therapy	Transcutaneous electrical acustimulation	Clinical (Human)	Bilateral ST36 (Zusanli) and bilateral PC6 (Neiguan) acupoints. Lasted for 4 weeks.	Patients diagnosed with GERD	Reduced postprandial indigestion symptoms.	Gastrointestinal, nervous system.	Stimulates the bilateral ST36 and PC6 points to activate the vagus nerve.	Vagus nerve, ST36 and PC6 points.	(25)
Neurostimulation Therapy	Transcutaneous abdominal electrical stimulation	Clinical (Human)	Applied to the abdominal area to stimulate muscular contractions. Lasted for 4 days.	Patients diagnosed with GERD, particularly those with resistance to PPI therapy	Reduced acid exposure time in PPI treatment-resistant GERD patients resulted in a reduction in DeMeester scores of more than 50%.	Gastrointestinal, nervous system.	Electrical stimulation regulates gastrointestinal motility and secretory function.	Gastrointestinal motor function, gastric acid secretion.	(25, 26)
Acupuncture Therapy	Wen’s modern scalp and auricular acupuncture	Clinical (Human)	Four treatments were conducted within two weeks	Patients with recurrent GERD symptoms who were dependent on PPIs for at least 6 months.	Regulated esophageal and gastric motility, visceral sensitivity, and esophageal epithelial barrier function.	Gastrointestinal, nervous system, immune system.	Regulates ANS, improves immune and barrier function of the esophagus, and reduces inflammatory response.	ANS, esophageal epithelial barrier, inflammatory cytokines.	(27)

## 3 Mechanisms underlying LES dysfunction in GERD

**Figure 1:**
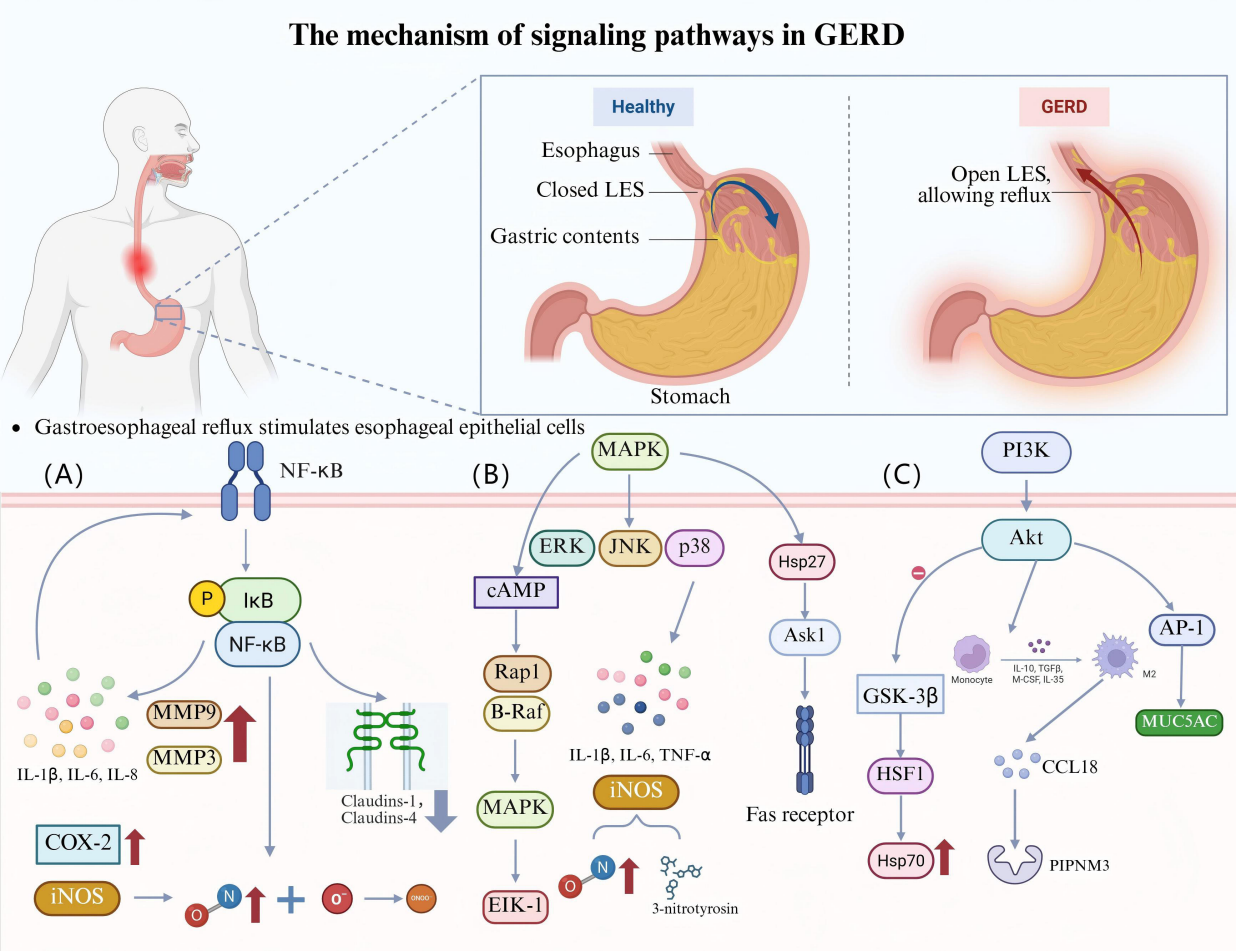
The roles of signaling pathways in GERD. **(A)** Esophageal epithelial cells are stimulated by gastroesophageal reflux, activate the NF-κB pathway, and upregulate the expression of MMP-3, MMP-9, IL-1β, IL-6, and IL-8, forming a positive-feedback loop. NF-κB also leads to the downregulation and mislocalization of claudin-1 and claudin-4, which increase esophageal epithelial permeability and exacerbate inflammation. In addition, NF-κB upregulates the expression of COX-2 and iNOS. iNOS overproduction yields NO, which reacts with O_2_^-^ to generate ONOO^-^, leading to DNA damage and participating in inflammation-related carcinogenesis. **(B)** The acidic environment activates the MAPK pathway (ERK, JNK, p38 MAPK) in EAC cells. TNF-α, IL-6, and IL-1β are released when p38 MAPK is activated, aggravating inflammation in the esophageal mucosa, and the expression of iNOS is simultaneously upregulated, which induces the production of NO and 3-nitrotyrosine, increasing oxidative stress and damaging the esophageal mucosa. cAMP activates MAPK and its downstream transcription factor Elk-1 through B-Raf- and Rap1-dependent pathways, and synergistically promotes BE cell proliferation and chemotaxis with MAPK. HSP27 regulates Ask1 activity and Fas receptor function, affects apoptosis, and may promote abnormal cell proliferation and malignant transformation. **(C)** Acid reflux activates the PI3K/Akt pathway, inhibits GSK-3β, regulates HSF1 activity, and promotes HSP70 expression. Bile acids activate the PI3K/Akt pathway by regulating EGFR expression in exosomes, inducing macrophage M2 polarization, releasing CCL18 to bind to PITPNM3, and promoting EAC cell proliferation. In addition, bile acids regulate MUC5AC expression through the PI3K/Akt/AP-1 pathway, and MUC5AC is significantly expressed in BE and EAC.

The main function of the LES, which is situated at the esophagogastric junction (EGJ), is to maintain a resting pressure higher than the intra-abdominal pressure. This difference in pressure prevents the stomach contents from refluxing into the esophagus, thereby avoiding irritation of the esophageal mucosa by stomach acid and the resultant discomfort (28). The diaphragm and esophageal hiatus contract during inspiration or periods of elevated intra-abdominal pressure, increasing the pressure in the EGJ and fortifying the anti-reflux barrier’s function (29). The main mechanisms underlying GERD include episodes of transient lower esophageal sphincter relaxation (TLESR) and decreased LES pressure (30, 31). The LES is a key component in maintaining the high-pressure reflux barrier, which consists of the LES, esophagogastric angle, phrenic pedicle, and phrenoesophageal ligament. Over time, GERD may develop as a result of changes to the reflux barrier’s structure and function (32) (**Figure 1**).

Studies have shown that absolute resting LES pressure cannot reliably predict the presence of GERD, but it can be used to measure the severity of GERD. In contrast, the relative LES pressure has been shown to be an essential predictor of the presence and severity of GERD. The transdiaphragmatic pressure gradient (TPG) has been shown to be higher in patients with GERD-positive and is strongly correlated with the presence and severity of GERD. An increase in the TPG is mainly associated with increased abdominal pressure rather than changes in chest pressure, which further highlights the essential role of abdominal pressure in the pathophysiology of GERD (33). Patients with GERD require customized management, and high-resolution manometry can serve as an effective tool for managing these patients by accurately evaluating the esophageal motility function and the status of the esophagogastric junction (34). More recently, anti-reflux mucosal resection (ARMS), an emerging treatment modality, has shown promising efficacy in patients with PPI-refractory GERD. ARMS has been shown to significantly improve the symptoms of patients with GERD by reducing the frequency of reflux episodes, shortening the duration of acid reflux into the esophagus, reducing the quantity of TLESRs, and decreasing the sensitivity of gastric tug receptors (8). Moreover, lower esophageal sphincter electrical stimulation (LES-EST) has been identified as a new technique for treating GERD. It has been shown to be safe and effective with no effect on swallowing function in patients with GERD combined with ineffective esophageal motility (IEM) (9) (**Table 1**).

## 4 Roles of immune cells and cytokines in GERD

The inflammatory response in GERD was traditionally thought to result from direct damage to esophageal epithelial cells by gastric acid and pepsin, known as the “acid burn model.” However, recent studies have proposed the “cytokine sizzle model,” which emphasizes the central role of immune cells and the cytokines they secrete in the inflammatory process of GERD (35). This new understanding provides a more comprehensive explanation for the pathogenesis of GERD and lays the theoretical foundation for the creation of immune-system–targeting treatment approaches (**Figure 1**).

### 4.1 T-lymphocytes

T-lymphocytes are a key immune cell type responsible for cellular immunological responses, and they play crucial roles in the infiltration of the esophageal epithelium and the inflammatory response in patients with GERD. Approximately 14% of patients with GERD exhibit lymphocytic inflammation of the esophageal epithelium, with 5.6% of these patients showing scattered lymphocytic infiltration associated with RE (36). In a rat model of RE, infiltration of T-lymphocytes was shown to be a key driver of the early inflammatory response. By postoperative day 3, T-lymphocytes had begun infiltrating the esophagus’s submucosal layer. Over time, these cells spread into the lamina propria during week one and into the epithelial layer during week three. Notably, T-cell infiltration was followed by surface cell injury and basal cell growth. Thus, inflammation is primarily driven by an immune response rather than being caused solely by direct damage from acids (37). This finding challenges the conventional wisdom that acid reflux causes an inflammatory reaction from cell death and granulocyte infiltration by disrupting the connective structures of esophageal epithelial cells, leading to acid infiltration into the cells.

The infiltration patterns of T-lymphocytes and their subpopulation distributions show notable differences across different stages of GERD. In patients with acute GERD, after cessation of PPI therapy, esophageal inflammation was predominantly characterized by a predominance of T-lymphocyte infiltration, with minimal involvement of neutrophils and eosinophils. In addition, these patients showed basal cell proliferation in areas without surface erosion (38). As GERD progresses, the pattern of T-lymphocyte infiltration changes. In patients with erosive esophagitis (EE), the proportion of T-lymphocytes in the esophageal squamous epithelium was much higher than that in patients with NERD. Specifically, the fraction of CD8^+^ T cells increased dramatically as the severity of GERD increased. When GERD progresses to BE, the inflammatory response shifts from a predominantly cellular immune response to a predominantly humoral immune response. T-lymphocyte reduction may inhibit the progression of GERD to BE, thereby reducing the risk of esophageal adenocarcinoma (EAC) (39, 40). The number of regulatory T cells (Tregs) has been shown to be increased in patients with GERD, which may suppress the inflammatory response caused by acid reflux. However, Treg function and quantity may not be sufficient to completely inhibit the inflammatory response (41). In GERD, lymphocytic inflammation primarily manifests as lymphocytic esophagitis (LyE). This process is characterized by a significant increase in the number of peripheral lymphocytes in the esophageal epithelium, whereas granulocytes are rare or absent. The high percentage of CD8^+^ T cells in LyE may help distinguish LyE from other types of esophageal inflammation (36).

**Figure 2:**
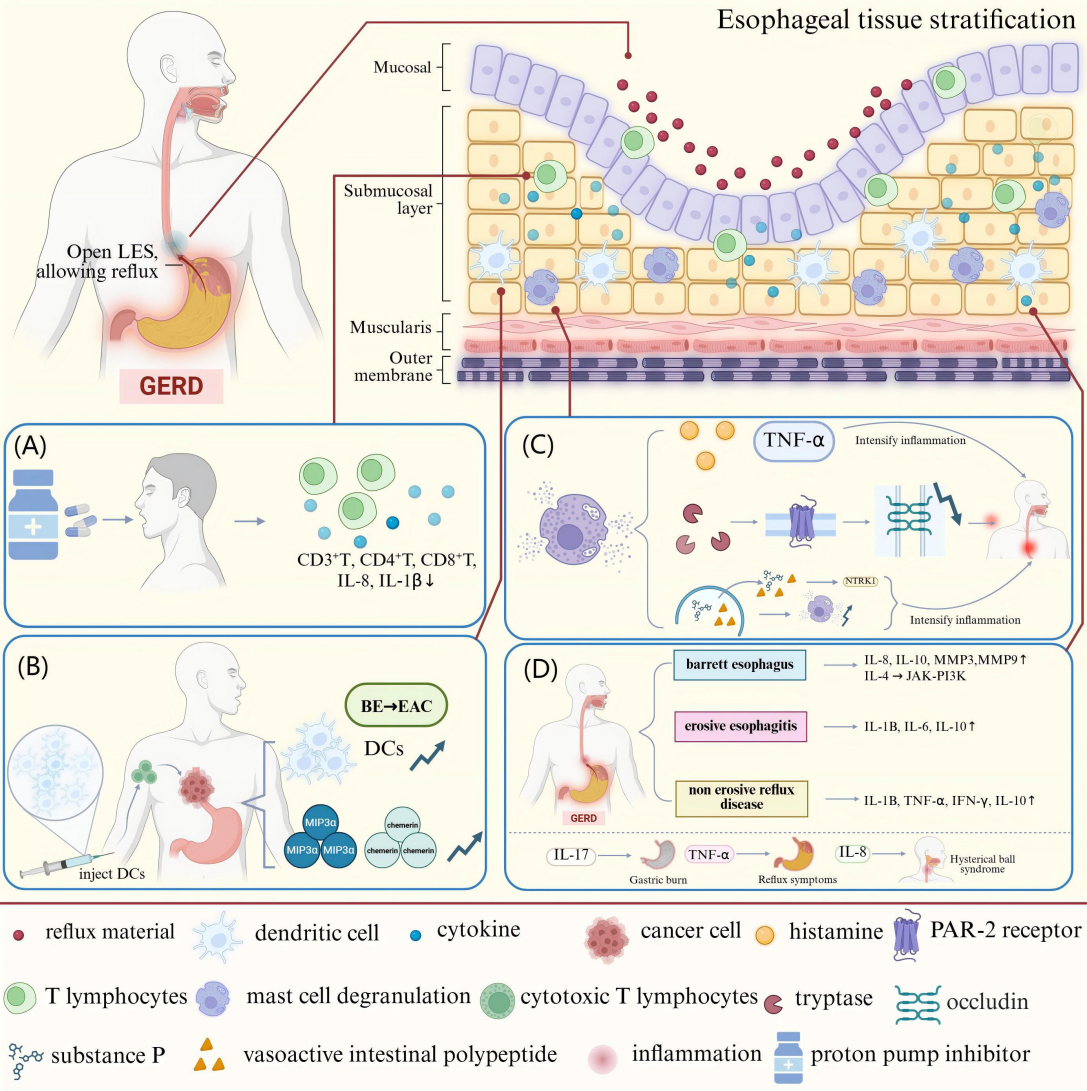
The mechanisms underlying the effects of immune cells and cytokines in GERD. **(A)** T-lymphocytes infiltrate all layers of the esophageal epithelium in patients with GERD. After PPI treatment, the CD3^+^, CD4^+^, and CD8^+^ T-cell counts in the peripheral blood decline along with the levels of the pro-inflammatory cytokines IL-8 and IL-1β. **(B)** The conversion of BE to EAC is accompanied by a reduction in the number of DCs in tissues. DCs can be used as immunotherapeutic targets by loading tumor-associated antigens and CTLs to attack tumor cells. **(C)** Degranulation of MCs releases pro-inflammatory mediators such as TNF-α, histamine, and trypsin-like enzymes. The activation of PAR-2 by trypsin-like enzymes reduces occludin expression and expands cell gaps, destroying the esophageal mucosal barrier. MCs can also release SP, VIP, etc., which act on the NTRK1 receptor on sensory neurons, triggering the nociceptive allergy and inflammatory response of the esophagus. **(D)** Patients with distinct GERD phenotypes show variable levels of inflammatory mediator expression in the esophageal mucosa. MMP-3, MMP-9, IL-8, and IL-10 expression are all markedly elevated in patients with BE, and IL-4 activates the JAK-PI3K signaling pathway in these patients to cause differentiation of the esophageal squamous epithelium into columnar epithelium. The expression of IL-1β, IL-6, and IL-10 is markedly elevated in patients with EE. Patients with NERD show higher levels of IL-1β, TNF-α, INF-γ, and IL-10 expression. Among symptoms, heartburn is mainly mediated by IL-17, TNF-α mediates reflux, and globus pallidus is mainly mediated by IL-8.

In a study on patients with GERD receiving PPIs, 66% of patients showed a significant decrease in T-cell counts following treatment, but 33% of patients still showed a high density of activated T-cell infiltration. Furthermore, PPI treatment was followed by a substantial reduction in the total number of CD3^+^, CD4^+^, and CD8^+^ T cells in the peripheral blood. Additionally, the levels of interleukin (IL)-8 and IL-1β reduced, suggesting that PPI treatment may act through systemic immunosuppression. However, patients who did not show any improvement may have been resistant to inflammation due to persistent T-cell activation (10). This finding suggests that immune-modulation strategies targeting T cells may represent a new direction for treating GERD (**Table 1**, **Figure 2**).

### 4.2 Dendritic cells

Human dendritic cells (DCs) are mainly divided into two subgroups: myeloid DCs (mDCs) and plasmacytoid DCs (pDCs). During the course of GERD, patients’ esophageal mucosa shows much fewer DCs than healthy tissues. Notably, DCs perform dual functions in healthy esophageal tissues: they are responsible for monitoring pathogens and suppressing adaptive immune responses by releasing anti-inflammatory cytokines. Therefore, a reduction in the number of DCs may cause pathologic alterations in the esophageal mucosa of patients and may result in abnormal activation of adaptive immune responses (42).

BE, as an intestinal epithelialized lesion of the distal esophageal squamous epithelium, is a precancerous lesion of EAC, with malignant transformation occurring in approximately 0.5–1% of patients each year (42). Using immunohistochemistry and electron microscopy techniques, one study confirmed for the first time the presence of DCs in BE and EAC tissues (43). Subsequent studies revealed that the number of mDCs in BE lesions was lower than that in normal esophageal tissues. However, the infiltration of mDCs increased significantly with the progression of BE to EAC. Meanwhile, the number of pDCs also showed a rising trend during the transformation of BE to EAC. This phenomenon may be related to the high expression levels of the chemokines macrophage inflammatory protein (MIP)-3α and chemerin. Notably, mDCs co-cultured with BE and EAC cell lines exhibited a tolerogenic profile, as evidenced by increased secretion of IL-10 and decreased secretion of IL-12p70. This cytokine secretion profile may promote the production of Tregs, suppressing anti-tumor immune responses (44). In patients showing progression to EAC, the function of DCs may be affected by various factors. For example, in patients with BE, upregulation of the exogenous metabolic pregnane X receptor signaling pathway is associated with exposure to harmful substances such as bile acids, which may lead to mucosal and DNA damage. This environment may inhibit the function of DCs, thereby impairing immune surveillance (45).

Due to their vital function in the immunological milieu of EAC, DCs have become viable targets for immunotherapy. Modifying DC activity to boost anti-tumor immune responses could lead to new treatment approaches to stop the progression of the disease (46). At present, the application of DCs in EAC therapy is mainly reflected in the use of DCs as a carrier of immunotherapy to attack tumor cells by loading tumor-associated antigens (TAAs) to activate specific T-cell responses, especially cytotoxic T-lymphocytes (CTLs). To improve the anti-tumor immune response, DC immunotherapy can be used in conjunction with chemotherapy, radiation therapy, or other targeted therapies such as anti-vascular endothelial growth factor (VEGF) and anti-human epidermal growth factor receptor (HER)-2/neu medications. For example, the use of anti-HER-2/neu antibodies (e.g., trastuzumab) in combination with DCs can significantly enhance the HER-2-specific CTL response, thereby improving tumor cell clearance (11) (**Table 1**, **Figure 2**).

### 4.3 Mast cells

The skin, respiratory system, and digestive system—all of which come into contact with the outside world—are home to a large number of mast cells (MCs), which are crucial cells in the immune system (47). The esophageal mucosa in patients with NERD shows a noticeably higher amount of MCs (48). Acid reflux can activate MCs, leading to degranulation and the production of pro-inflammatory mediators like histamine and tumor necrosis factor (TNF)-α, which further impair the esophageal mucosa’s barrier function and intensify the inflammatory response. Activated MCs release trypsin-like enzymes, an important inflammatory mediator. These enzymes cause a pro-inflammatory reaction by activating proteinase-activated receptor 2 (PAR-2). The resultant inflammatory response in esophageal epithelial cells can reduce the expression of tight junction proteins like occludin and widen the cellular gap, impairing the esophageal mucosa’s barrier function (49). A bidirectional nerve-MC signaling network is formed by the close contact between MCs and esophageal nerve fibers. In GERD, MCs act on receptors on sensory neurons by releasing neuropeptides, such as substance P (SP) and vasoactive intestinal polypeptide (VIP), and pro-inflammatory mediators, such as neurotrophic tropomyosin receptor kinase 1 (NTRK1), drive neuronal plasticity and peripheral sensitization, leading to esophageal nociceptive hypersensitivity and inflammatory responses. This mechanism is particularly prominent in patients with NERD (50, 51). In addition, neuropeptides released in neurogenic inflammation, such as SP and VIP, may promote the degranulation of MCs, resulting in a positive-feedback loop that causes worsening of symptoms (52). 5-Hydroxytryptamine (5-HT) released from MCs is essential for controlling the gastrointestinal tract’s motility and sensory processes. 5-HT, through activation of 5-HT3 receptors, affects the esophageal response to distension sensitivity and visceral perception, which may be related to the visceral hypersensitivity in patients with GERD (53). Estrogen has been shown to reduce RE-induced esophageal damage by preventing MCs from expressing TNF-α. The lack of protective effects of estrogen in men may result in an increased prevalence of GERD, which may be one of the essential mechanisms underlying the sex-related differences in GERD (54). Research has shown that patients with eosinophilic esophagitis (EE) have an increased number of mast cells in their esophagus and elevated levels of mast cell degranulation. These cells release pro-inflammatory mediators such as trypsin and carboxypeptidase A3, which play an important role in it. In addition, KIT ligands specifically involved in mast cells are considered potential targets for treating EE (55). MC activates PAR-2 and vasoactive intestinal peptide receptor (VPAC-1) by releasing pro-inflammatory mediators, thereby enhancing neural signaling and inflammatory response (56). Meanwhile, MC is also the main source of LI-13 mRNA and protein in the esophagus, while IL-13 is considered a core pro-inflammatory factor in the pathogenesis of EE. By inhibiting IL-13 or affecting the activation and migration of MC, the inflammatory response of EE can be effectively alleviated (57).

Hewei Jiangni granule (HWJNG) was found to show promise as a therapeutic agent for NERD. HWJNC inhibited the degranulation of MCs in the serum and esophageal tissues of rats with NERD, decreased the expression of trypsin-like enzymes, modulated the mRNA and protein levels of transient receptor potential vanilloid 1 (TRPV1) and transient receptor potential melastatin channel subfamily member 8 (TRPM8), and reduced the expression of neurochemicals such as SP, calcitonin gene-related peptides (CGRP), and proteinase-activated receptor 2 (PAR2), thereby exerting anti-inflammatory and analgesic effects (12). In addition, Jianpi Qinghua (JQ) granules caused a notable decrease in reflux and heartburn symptoms, improved GERD health-related quality of life (HRQL), and decreased self-reported anxiety and sadness scores in patients with NERD. In GERD models, JQ granules have been shown to improve mucosal barrier integrity, lessen nerve-ending sensitization, and encourage the growth and activation of MCs in the esophageal mucosa (13). The protective effects of estrogen and the potential efficacy of herbal therapy provide new research directions for the treatment of GERD (**Table 1**, **Figure 2**).

### 4.4 Mechanisms underlying the effects of cytokines in GERD

During the pathophysiology of GERD, multiple cytokines interact to form a complex inflammatory network, and these cytokines play essential roles in esophageal mucosal injury, repair, and immunomodulation (38). The release of IL-8 and IL-1β has been shown to be considerably enhanced in esophageal epithelial cells exposed to acidic bile salts. These pro-inflammatory cytokines can attract immune cells (e.g., T cells and neutrophils) to migrate toward esophageal tissues, triggering a localized inflammatory response. Usually starting in the submucosal layer, this inflammatory process progressively spreads to the epithelial layer before causing erosions and ulcers on the surface of the esophagus (37). Notably, IL-8 levels were found to decrease after PPI treatment and anti-reflux surgery, which further confirmed the crucial role of IL-8 in mucosal injury (58).

Inflammatory mediators have been shown to be differently expressed in the endoscopic esophageal mucosa of individuals with various GERD phenotypes. In patients with BE, the expression of IL-8, IL-10, matrix metalloproteinase (MMP)-3, and MMP-9 was found to be significantly upregulated. Notably, by triggering the Janus kinase (JAK)-PI3K signaling pathway, IL-4 caused esophageal squamous epithelial cells to differentiate into columnar epithelial cells. This process has been shown to significantly contribute to the development of BE (59). Patients with EE show a considerable increase in the expression of IL-1β, IL-6, and IL-10, and their inflammatory response shows a dynamic balance of Th1 and Th2 cytokines. In patients with NERD, the expression of IL-1β, TNF-α, interferon (INF)-γ, and IL-10 was increased in the abnormal acid-exposed group (60). Subsequent research revealed a clear correlation between the duration of acid exposure and pH and the gene expression levels of inflammatory cytokines in the esophageal mucosa of patients with GERD. Specifically, esophageal acid exposure time was positively correlated with gene expression levels of IL-1β, IL-18, TNF-α, CD68, and β2 microglobulin (B2M). The mean pH of the lower esophagus was negatively correlated with the expression levels of IL-18, TNF-α, GATA binding protein 3 (GATA3), Toll-like receptor 4 (TLR4), and CD68 (61). It should be noted that the expression of IL-33 is significantly upregulated in GERD. IL-33, as a tissue derived cytokine, is mainly expressed in the basal cell layer of the esophagus and drives inflammatory responses through its receptor ST2. It is significantly correlated with the expression of cytokines such as IL-6 and IL-8. In addition, the upregulation of IL-33 is closely related to the increase of intercellular space (ICS), suggesting that it may exacerbate the damage of gastric acid and other refluxes to the esophageal mucosa by disrupting the epithelial barrier function (62). Different symptom phenotypes of GERD have been associated with distinct expression patterns of specific cytokines. IL-17 mainly mediates heartburn symptoms and may be related to activation of acid-sensitive receptors and pro-inflammatory responses. TNF-α mediates reflux symptoms and may be associated with esophageal sphincter function and mechanical processes. In contrast, dysthymia is mainly mediated by IL-8, which may be linked to extraesophageal symptoms. These findings reveal the heterogeneity of GERD symptoms and provide a conceptual framework for developing targeted treatments that focus on specific cytokines. However, existing studies still have some limitations, such as the absence of a robust control group and detailed pH monitoring data (63). At the level of molecular mechanisms, the expression of several important inflammatory mediators, including nitric oxide synthase (NOS), myeloperoxidase (MPO), and hypoxia-inducible factors, was shown to be considerably elevated in the esophageal mucosa of patients with GERD (64).

On the basis of the mechanistic studies described above, therapeutic strategies targeting inflammatory and immunomodulatory factors may provide new therapeutic approaches to improve GERD in patients who have failed PPI therapy. Berberine (BB) was found to exhibit low cytotoxicity and markedly reduce serum TNF-α, IL-1β, IL-6, and monocyte chemoattractant protein (MCP)-1 levels by activating AMP-activated protein kinase (AMPK) and inhibiting inflammatory cytokines (14). In another study, increasing the intake of fermented soybean (FSB) for three months significantly improved patients’ stomach burning sensation, which is possibly connected to the anti-inflammatory effects of the bioactive peptides in FSB. FSB not only significantly reduced the reflux symptoms, especially in elderly patients, but also lowered the levels of IL-8, IL-6, and IL-4, which are cytokines linked to the inflammatory response in GERD and are closely related (15). These results provide important clues for the development of innovative therapeutic agents for GERD (**Table 1**, **Figure 2**).

## 5 Mechanisms of signaling pathways in GERD

In the pathogenesis of GERD, the aberrant activation of multiple signaling pathways creates a positive-feedback loop that exacerbates esophageal inflammation and barrier dysfunction. Specific suppression of certain signaling pathways (e.g., using nuclear factor [NF]-κB inhibitors, cardiac glycosides, or Chinese herbal medicine combinations) can lessen the inflammatory response and repair the mucosal barrier, offering a fresh approach to treating GERD. Meanwhile, the interactions among these pathways can also reveal the potential mechanism of GERD progression to BE and EAC. However, most of the current evidence relies on animal models, which may not fully capture the heterogeneity of human GERD. In the future, randomized clinical trials and the development of advanced human models (such as patient derived organoids) will be needed to validate these findings.

### 5.1 NF-κB signaling pathway

Gastroesophageal reflux directly activates the NF-κB pathway by stimulating esophageal epithelial cells. This process is accompanied by upregulation of MMP-3, MMP-9, IL-1β, IL-6, and IL-8. Expression of NF-κB by the esophageal epithelium is further activated by the increased expression of these inflammatory substances and inflammatory cells, creating a positive-feedback loop. In addition, over time, the expression of these cytokines leads to downregulation and mislocalization of the tight junction proteins claudin-1 and claudin-4, which increases esophageal epithelial permeability and exacerbates the inflammatory response. Studies have shown that this process can be blocked using the NF-κB inhibitor BAY 11-7085, protecting esophageal barrier function and attenuating the inflammatory response (65, 66). Another study showed that reflux stimulates the esophageal mucosa and activates the TLR4/NF-κB pathway, causing oxidative stress and an inflammatory response, which induces esophageal mucosal injury. Activation of the TLR4/NF-κB signaling pathway promotes apoptosis and aggravates the damage to the esophageal mucosa by modulating the expression of Bax, Bcl-2, and caspase-3. In this regard, blocking the TLR4/NF-κB pathway has been shown to attenuate inflammatory responses, reduce oxidative stress, repair mucosal barrier function, and inhibit apoptosis (67). In addition, activation of NF-κB results in the release of the cytokines TNF-α and IL-6. By modifying the expression of cyclooxygenase-2 (COX-2), NF-κB controls the expression of COX-2 and inducible NOS (iNOS), which is enhanced under inflammatory conditions such as RE and BE. Meanwhile, the expression of iNOS leads to the overproduction of nitric oxide (NO). The reaction of NO with superoxide anion (O_2_^-^) generates peroxynitrite (ONOO^-^), which induces DNA damage and participates in inflammation-associated carcinogenesis (16) (**Figure 1**).

Herbal therapy has been utilized extensively to treat GERD. Rhei Rhizoma has shown the ability to disperse blood stasis, activate circulation, expel heat, and extinguish fire (68). Rhei Rhizoma has also been shown to reduce the generation of reactive oxygen species (ROS) and alleviate oxidative stress by activating the nuclear factor erythroid 2-related factor 2/heme oxygenase-1 (Nrf2/HO-1) pathway. In addition, Rhei Rhizoma can inhibit the release of inflammatory mediators by inhibiting the MAPK signaling pathway, blocking the phosphorylation of IκBα, and preventing the nuclear translocation of NF-κB (16). Geraniin is obtained from geraniums. The main polyphenolic compounds in geraniums possess a range of pharmacological actions, including anti-inflammatory and antioxidant qualities (17). Among these, geraniin can attenuate lipopolysaccharide (LPS)-induced macrophage inflammatory responses and exert antioxidant activity by inhibiting NF-κB and Nrf2 (18). Quercetin is a naturally occurring polyphenolic molecule with a number of antiviral, anti-inflammatory, immunomodulatory, and antioxidant properties (19). In RE rats, quercetin has been shown to successfully stop esophageal mucosal damage by blocking the NF-κB p65 and IL-8 signaling pathways (20) (**Table 1**).

### 5.2 MAPK signaling pathway

The MAPK signaling pathway consists of three major subfamilies: extracellular signal-regulated kinase (ERK), c-Jun N-terminal kinase (JNK), and p38 MAPK (69). Transient exposure to an acidic environment has been shown to induce activation of the MAPK pathway in EAC cells *in vitro* (70). Fourteen days after esophageal mucosal damage, the p38 MAPK signaling pathway was markedly activated in the esophageal mucosa of RE rats. Conversely, the function of the esophageal barrier was improved by inhibition of p38 MAPK, which also increased the expression of tight junction proteins and decreased the expression levels of MMP-3 and MMP-9 (71). Activation of the p38 MAPK signaling pathway released the pro-inflammatory cytokines TNF-α, IL-6, and IL-1β in the RE, aggravating the inflammatory response in the esophageal mucosa and causing tissue damage (72). p38 MAPK suppression was shown to dramatically reduce the expression of TNF-α, IL-6, and IL-1β and decrease the infiltration of CD68-positive cells in the esophageal mucosa (71). Furthermore, p38 MAPK activation has been shown to enhance iNOS expression, which results in the synthesis of NO and 3-nitrotyrosine. This exacerbates oxidative stress and further harms the esophageal mucosa (72). Cyclic adenosine monophosphate (cAMP) is an essential second messenger with tissue-specific effects on cell growth, differentiation, and gene expression. cAMP can activate MAPK and its downstream transcription factor Elk-1 through B-Raf- and Rap1-dependent pathways. Research has shown that the cAMP and MAPK signaling pathways may synergistically act during cell proliferation and chemotaxis in BE (70). Furthermore, in esophageal endothelial cells exposed to acidic environments, heat shock protein 27 (HSP27) is a crucial cellular defense protein that shields the esophagus from different cytotoxic insults and promotes tissue recovery. HSP27 controls apoptosis via influencing the function of the Fas receptor, a crucial cell surface death receptor, and by modifying the activity of apoptosis signal-regulating kinase-1 (Ask1), a member of the MAPK family. This regulation may perform a dual role, protecting cells from damage and also promoting aberrant cell proliferation by inhibiting apoptosis, increasing the risk of malignant transformation (73) (**Figure 1**).

Recent studies have shown that the levels of MAPK kinase 6 (MKK6), a key upstream regulator of the MAPK pathway, are elevated in EAC. Cardiac glycosides such as ouabain, digoxin, and digitoxin have shown the ability to downregulate MKK6 expression and inhibit the growth of EAC cells (74). Furthermore, a traditional Chinese medicine compound named Zuojin Pill (ZJP) has been studied recently for the treatment of GERD. ZJP inhibits the activation of the MAPK/NF-κB signaling pathway by decreasing the phosphorylation levels of p65, JNK, and ERK1/2. This pathway drives the release of inflammatory mediators in GERD, and the inhibitory effects of ZJP reduce the production of TNF-α, IL-6, and IL-1β downstream of the MAPK/NF-κB signaling pathway, thereby attenuating the inflammatory response (21). In addition to participating in controlling the inflammatory response in GERD, BE, and EAC, the MAPK signaling pathway also influences processes such as apoptosis, proliferation, and oxidative stress, which drive disease progression. Interventions targeting the MAPK pathway, such as the use of cardiac glycosides or drugs such as ZJP, may provide new strategies for treating GERD and related diseases (**Table 1**).

### 5.3 PI3K-Akt signaling pathway

The PI3K/AKT pathway causes esophageal mucosal injury in GERD by increasing the expression of inflammatory factors and promoting oxidative stress (75). Acidic reflux (pH 4.5) has been shown to significantly enhance the expression of HSP27 and heat shock protein 70 (HSP70) in human esophageal microvascular endothelial cells, which may have a protective effect on the cells. By triggering the PI3K/Akt signaling pathway and blocking glycogen synthase kinase-3β (GSK-3β), acid exposure also controls the activity of heat shock transcription factor-1 (HSF1), which in turn increases the expression of HSP70 (76). Acidic bile salts have also been shown to activate the PI3K/AKT signaling pathway and control the expression of the epidermal growth factor receptor (EGFR) in exosomes, which in turn causes macrophage M2 polarization. The cytokine CCL18 released by M2 macrophages binds to its receptor PITPNM3, promoting the proliferation of EAC cells (77). Additional studies revealed that unbound bile acids induced CRE binding protein and activator protein-1 (AP-1)-dependent COX-2 expression in BE and EAC through ROS-mediated activation of PI3K/AKT and ERK1/2, thereby promoting EAC development (78). This mechanism highlights the important role of bile acids in esophageal carcinogenesis. Another study found that bile acids regulate mucin 5AC (MUC5AC) expression through the PI3K/AKT/AP-1 pathway. MUC5AC expression was more effectively increased at the transcriptional level by bound bile acids than by unbound bile acids. BE and EAC exhibited high levels of MUC5AC expression, but normal esophageal squamous epithelium did not (79).

Studies have shown that atractylenolide III (ATL III) improves RE and reduces oxidative stress and inflammatory responses by inhibiting the PI3K/AKT/NF-κB/iNOS signaling pathway (22). In addition, the traditional Chinese medicine compound Zhizhu pill (ZZP) has been shown to alleviate GERD through multi-component and multi-target properties. Network pharmacological analyses indicated that ZZP improves the symptoms of GERD by inhibiting the PI3K/AKT pathway (23). The expression level of phosphatidylinositol 3-kinase regulatory subunit beta (Pik3r2), a crucial protein in the PI3K/Akt signaling pathway that is intimately linked to neuronal proliferation, survival, synaptic plasticity, and cognitive function, can be decreased by the Chinese herbal formula Shugan Jiangni Hewei granules (SJHG). SJHG may exert neuroprotective effects by regulating this pathway (24) (**Table 1**, **Figure 1**).

## 6 Mechanisms of the microbiota-gut-brain axis in GERD

The microbiota-gut-brain axis (MGBA) has garnered much interest in recent research on GERD. The microbiota encompasses the complex community of microorganisms that inhabit the gastrointestinal tract. It is increasingly recognized as a vital regulator of host physiology, influencing immune responses, neural signaling, and metabolic processes (80). Individuals with GERD often show dysbiosis of the intestinal flora, abnormal neuromodulation, hormone secretion disorders, and abnormal immune responses, which interact with each other through the MGBA and may affect the motility and secretion functions of the gastrointestinal tract to aggravate reflux symptoms. The MGBA-targeting therapeutic strategies for GERD focus on reshaping the homeostasis of the gut microbiota, promoting the proliferation of beneficial bacteria and the inhibition of harmful bacteria, improving the functioning of the neuro-endocrine-immune system, and relieving psychological stress. The MGBA targeted therapy strategy for GERD focuses on reshaping the composition and diversity of the gut microbiota, promoting the growth and proliferation of beneficial bacteria, while inhibiting the overgrowth of harmful bacteria. Enhance neuroendocrine signaling pathways, regulate inflammatory cytokines, and promote immune homeostasis. The psychological factors of GERD can also be addressed by reducing the hypothalamic-pituitary-adrenal (HPA) axis.

### 6.1 Composition and function of the MGBA

**Figure 3:**
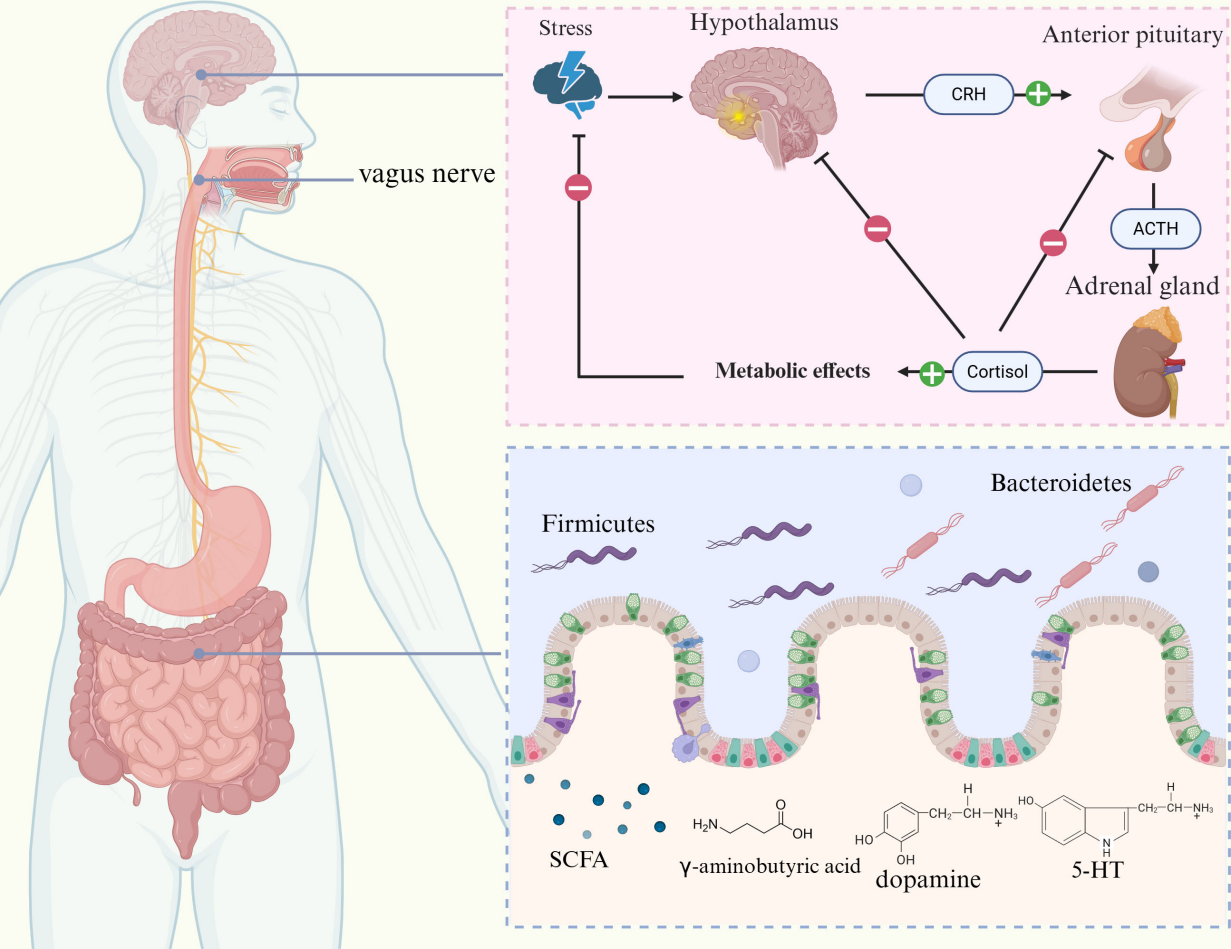
Relationship between the gut-brain axis and GERD. The gut-brain axis consists of the CNS, ENS, ANS, HPA axis, and gut microbiota. CRH, ACTH, and cortisol are released by the HPA axis, which controls the body’s stress response. Elevated cortisol levels can increase intestinal permeability and disrupt the intestinal barrier function. Furthermore, the gut microbiota plays a role in regulating mood and homeostasis of the gut environment through bidirectional communication with the brain through the secretion of SCFAs, 5-HT, dopamine, and γ-aminobutyric acid.

The MGBA refers to the bidirectional communication pathway linking gut microorganisms with the central nervous system, involving the central nervous system (CNS), enteric nervous system (ENS), autonomic nervous system (ANS), HPA axis, and the gut microbiota (81, 82). The ENS has been suggested to regulate gut function independently and interact closely with the CNS. Due to local inflammation and intestinal dysfunction, the incoming nerves of the gastrointestinal tract are overactivated, leading to visceral hypersensitivity reactions. Multiple molecular mechanisms are involved in this process, including the TRPV family, serotonin receptors, protease activated receptors (PARs), and cannabinoid receptors, which stimulate the release of acetylcholine and substance P by binding to endogenous ligands such as gut hormones and immune mediators, promoting pain signal transduction. At the same time, the processing centers of the spinal cord and CNS are more sensitive to peripheral signals, leading to excessive amplification of pain signals (83). In the case of MGBA dysfunction, the vagus nerve’s ability to control visceral perception decreases, leading to ineffective activation of the descending inhibitory pathway in the brainstem, thereby weakening its inhibitory effect on pain signals (25). In addition, MGBA dysfunction further damages the neural networks of ENS and CNS through maladaptive neuroplasticity induced by chronic stress (84). Pressure affects the composition of the gut microbiota through the HPA axis, particularly with changes in the ratio of Bacteroidetes and Firmicutes. The number of Bacteroidetes in depressed individuals is usually higher, while the proportion of Firmicutes is lower. Transferring the fecal microbiota of depressed individuals to healthy animals has been shown to induce depression-like behaviors in recipient animals (85). The MGBA regulates gut-brain interactions through neural, endocrine, and immune pathways, affecting appetite, metabolism, and feeding behavior (82, 86). Additionally, the gut microbiota affects the function of the HPA axis by releasing corticotropin-releasing hormone (CRH), adrenocorticotropic hormone (ACTH), and cortisol, which regulate the basic physiological states of the brain. The gut microbiota also maintains gut stability by producing short-chain fatty acids (SCFAs) as well as neurotransmitters (e.g., 5-HT, dopamine, γ-aminobutyric acid) which facilitate bidirectional communication with the brain (87). Complex emotional stimuli are processed via the thalamo-cortico-amygdala pathway, involving regions such as the anterior cingulate cortex and the prefrontal cortex (PFC) (88). Research has found that the abundance of Lactobacillus and Bifidobacterium significantly decreases in GERD patients (89). GERD patients typically require the use of gastric acid inhibitors, however, these drugs may disrupt the balance of gastrointestinal microbiota by reducing gastric acid levels, thereby increasing the risk of Clostridium difficile infection (90). In addition, studies have found a significant increase in the abundance of Proteobacteria in GERD patients, and Escherichia is an important member of the Proteobacteria phylum, which may play a role in the development of GERD (91). The ENS, on the other hand, connects bidirectionally to the brain via parasympathetic and sympathetic pathways, whereas central stress circuits, including the paraventricular nucleus of the hypothalamus, the amygdala, and periaqueductal gray matter, are responsible for generating stress responses (92). Various stressors, such as anger, fear, and painful stimuli, affect gastric function, and these effects have been shown to be related to the etiology of stomach problems such as GERD (93) (**Figure 3**).

### 6.2 Association of GERD with neurodegenerative and psychiatric disorders

Studies have shown notable genetic and biological links between GERD and a variety of neurodegenerative and psychiatric disorders. GERD and neurodegenerative and psychiatric conditions, including major depressive disorder, anxiety disorders, and Alzheimer’s disease, share genes and molecular pathways that were identified through bidirectional Mendelian randomization analyses and chained disequilibrium score regression (94). A meta-analysis further confirmed a strong correlation between psychosocial issues and GERD. Patients with GERD were 2.63 times more likely to have depression and 3.43 times more likely to experience anxiety disorders, whereas those with psychosocial problems were 2.23 times more likely to experience GERD, with such problems potentially influencing the development of the condition (95). Individuals with NERD specifically exhibit higher levels of anxiety and depression, which may play a role in influencing their symptoms. Psychological factors may promote acid reflux through mechanisms such as reduced pressure in the LES, altered esophageal motility, or elevated gastric acid secretion, and persistent reflux symptoms may further trigger anxiety and depression (96, 97). In comparison with the general population, patients with GERD experience more active neural responses in the brain when exposed to the same stimuli, indicating that chronic psychological stress increases the perception of esophageal pain through the MGBA. Chronic stress activates the HPA axis, leading to the release of cortisol, which increases intestinal permeability and disrupts the integrity of the intestinal barrier (98, 99). Salivary cortisol is an important biomarker of HPA axis function, which can reflect the functional status of MGBA and its response to stress (100). The gut microbiota is responsible for 90% of 5-HT production, and decreased 5-HT levels are associated with depression. 5-hydroxyindoleacetic acid (5-HIAA) is the main metabolite of 5-HT, and its level changes can reflect the bidirectional regulatory effect between gut microbiota and neurotransmitter metabolism (101). Additionally, the gut microbiota can also convert tryptophan into indole sulfate, a compound associated with anxiety (99). Furthermore, in patients with GERD, the esophageal mucosa produces elevated levels of pro-inflammatory cytokines, such as IL-6, IL-8, and TNF-α, which can enter the peripheral circulation and potentially influence the CNS, contributing to the development of anxiety and depression (102). In addition, fecal calprotectin, as an established biomarker of intestinal inflammation, can reflect dysbiosis of the gut microbiota (103) (**Figure 3**).

### 6.3 Potential therapeutic strategies to modulate the MGBA

Therapeutic approaches based on modulation of the MGBA are currently being explored, and the main therapeutic agents that are under investigation in these studies include motilin receptor agonists, ghrelin, and CCK1 receptor agonists. In addition, alteration of the gut microbiota by dietary changes, probiotics, or prebiotics may help improve the MGBA’s functioning, thereby alleviating GERD and its associated psychological co-morbidities (104). Although PPIs are the first-line treatment option for GERD, excessive use of PPIs may affect the stability of the gut microbiota, further influencing the MGBA. The use of PPIs increases intragastric pH, allowing more bacteria to colonize the stomach and small intestine and potentially leading to alterations in the composition of the gut microbiota (99). Prolonged dependence on PPIs may exacerbate anxiety and also affect the mental health of patients. Glutamate, the primary excitatory neurotransmitter in the CNS, has been shown to act through the MGBA. Glutamate influences gut-brain communication by affecting afferent fibers to transmit signals from the gut to the brain and efferent fibers to relay messages from the brain to the stomach, thereby controlling the secretory and motor functions of the gut (105). Among non-pharmacological treatments, transcutaneous electrical acustimulation (TEA) and transcutaneous abdominal electrical stimulation have demonstrated some efficacy in treating GERD. TEA to the bilateral ST36 and PC6 acupoints has been shown to increase gastric regulatory pacing activity and reduce postprandial dyspeptic symptoms. These effects are thought to be mediated through a vagal mechanism (25). Preliminary studies have also shown that percutaneous electrical abdominal stimulation significantly reduces the acid exposure time in patients with GERD resistant to PPI therapy, with the DeMeester scores reducing by more than 50% in these patients (26). Wen’s modern scalp and auricular acupuncture (WMA) has been shown to have potential benefits in regulating visceral sensitivity, esophageal and gastric motility, and esophageal epithelial barrier function. Additionally, it dramatically decreased inflammatory cytokine levels, which may enhance the esophageal barrier and immunological function by influencing the ANS (27). These non-invasive treatments offer new approaches to control GERD and enhance patients’ quality of life in general (**Table 1**).

## 7 Discussion

This study examines the pathogenesis of GERD, emphasizing the crucial roles of the LES, inflammatory response, signaling pathways, and MGBA in the development of this disease. GERD is not simply a pathological process caused by acid reflux, but is instead a complex disease with multifactorial interactions. These findings emphasize the importance of adopting a multifaceted approach to understanding and treating GERD, especially in cases where conventional therapies such as PPIs have not been effective. Approximately 45% patients with GERD continue to experience symptoms even after using a PPI (106).

The LES plays a key role in maintaining the anti-reflux barrier. When this barrier fails, it can lead to GERD and even result in complications such as esophagitis, BE, and EAC. Reflux is most strongly associated with the TLESR and reduction of LES pressure. Therefore, therapeutic programs targeting these two key factors have emerged as an important strategy for treating GERD. More recently, ARMS and LES-EST have shown promising results for treating refractory GERD (8, 9). However, existing treatment modalities still show some limitations. For example, the long-term efficacy and safety of ARMS and LES-EST require further investigation, and their clinical application needs to be supported by more long-term follow-up data. Thus, optimizing these therapeutic modalities to improve patients’ quality of life is an important direction for future research.

Recent studies outlining the “cytokine storm model” have challenged the traditional view of GERD as a disease caused only by acid-induced mucosal damage. T-lymphocytes, DCs, and MCs have been shown to be key players in the inflammatory reaction in the esophagus in patients with GERD. Infiltration of these cells and the release of cytokines such as IL-8, IL-1β, TNF-α can collectively lead to mucosal damage and worsening symptoms. In particular, DCs are essential to the immune microenvironment of BE and EAC. Future studies could explore methods to enhance tumor elimination by modulating the function of DCs, especially during the conversion of BE to EAC. Some important agents, such as HWJNG and JQ granules, have shown the potential to inhibit the degranulation of MCs and modulate neuropeptide release, contributing to the alleviation of GERD-related symptoms. Notably, patients with GERD with various characteristics showed notable variations in cytokine patterns. Detection of inflammatory mediators in the esophageal mucosa may provide the basis for early diagnosis of GERD and assessment of its severity. Targeting these immune pathways is expected to provide new therapeutic avenues for patients with GERD, especially in individuals who have ongoing inflammation in spite of acid suppression.

Multiple inflammatory signaling pathways, including NF-κB, MAPK, and PI3K/Akt are essential for regulating the pathological process of GERD. These signaling pathways can not only drive the inflammatory response but may also promote the development of more serious conditions like BE and EAC from GERD. Therefore, studies on inhibitors targeting these signaling pathways are of particular importance, since their findings will provide new directions for drug development for clinical applications. A combination of traditional herbal component therapies, especially natural compounds such as Rhei Rhizoma, geraniin, and quercetin, shows potential to modulate these signaling pathways (16–20). Chinese medicine combinations, such as ZJP and ZZP, can also show multi-targeting effects (21, 23). To further evaluate the potential applications of these herbal components and compounds in GERD, more comprehensive basic research is essential to better understand their specific mechanisms in signaling pathways.

The perception of esophageal pain in GERD patients is the result of complex interaction between CNS and PNS. Pain regulation disorders in CNS may lead to overreaction to normal stimuli, while abnormalities in PNS, such as increased reactivity of esophageal wall nerve endings, may exacerbate symptoms. In addition, the gut microbiota structure of GERD patients is significantly different from that of healthy individuals, especially with changes in the abundance of certain bacterial groups, such as Lactobacillus, Bifidobacterium, Escherichia, and Clostridium difficile. Supplementation with probiotics such as lactobacilli and bifidobacteria has been shown to improve symptoms of GERD (107). In addition to causing a variety of physical problems, GERD can cause major psychosocial issues such as anxiety, sadness, and sleep disturbances. Systemic disease-related physical or psychological stress may trigger the immune system, increasing the release of pro-inflammatory cytokines and thus having an increasingly pronounced effect on GERD (98). Thus, clinicians should consider the presence of another disorder when treating one of them to achieve a more integrated treatment outcome. The MGBA has been increasingly studied in the context of GERD, particularly in understanding the link between psychological stress and reflux symptoms. Patients with GERD often show higher levels of anxiety and despair, which may exacerbate patients’ symptoms through mechanisms such as increased visceral sensitivity and altered esophageal motility. Non-pharmacological interventions, including probiotics, dietary modifications, TEA techniques, and acupuncture, can serve as effective interventions to improve GERD symptoms by modulating the MGBA. In addition, individualized nutritional counseling and psychological support may help lessen issues and raise living standards.

This review summarizes the key roles of immune cells, cytokines, signaling pathways, and the MGBA in the pathogenesis of GERD. Despite some advancements in research on the pathophysiology of GERD, many challenges remain unresolved. Existing studies are mostly based on animal models and *in vitro* experiments, and the relatively few clinical studies have yielded some controversial findings. Moreover, GERD is highly heterogeneous, with differences in clinical manifestations, pathogenesis, and treatment responses among different patients, making the achievement of personalized and precise treatment a major challenge. Future studies should focus on translating these findings into clinical practice, with emphasis on developing personalized treatment strategies and new therapeutic targets based on individual patient characteristics. Additionally, the roles of genetic predisposition, environmental factors such as diet and obesity, and the long-term effects of emerging therapies require further investigation. The development of more accurate diagnostic methods and personalized therapeutic regimens will help improve the overall treatment of GERD, thereby enhancing patients’ quality of life.
